# A phase II study of ibrutinib in combination with rituximab-cyclophosphamide-doxorubicin hydrochloride-vincristine sulfate-prednisone therapy in Epstein-Barr virus-positive, diffuse large B cell lymphoma (54179060LYM2003: IVORY study): results of the final analysis

**DOI:** 10.1007/s00277-020-04005-6

**Published:** 2020-04-24

**Authors:** Sang Eun Yoon, Seok Jin Kim, Dok Hyun Yoon, Youngil Koh, Yeung-Chul Mun, Young Rok Do, Yoon Seok Choi, Deok Hwan Yang, Min Kyoung Kim, Gyeong-Won Lee, Cheolwon Suh, Young Hyeh Ko, Won Seog Kim

**Affiliations:** 1 Division of Hematology and Oncology, Department of Medicine, Samsung Medical Center, Sungkyunkwan University School of Medicine, 81, Irwon-ro, Gangnam-gu, Seoul, 06351 South Korea; 2 Department of Oncology, Asan Medical Center, Ulsan University College of Medicine, Seoul, South Korea; 3 Department of Internal Medicine, Seoul National University Hospital, Seoul, South Korea; 4 Department of Hematology-Oncology, Ewha Womans University Mokdong Hospital, Seoul, South Korea; 5 Department of Hematology-Oncology, Keimyung University School of Medicine, Daegu, South Korea; 6 Department of Internal Medicine, Chungnam National University College of Medicine, Daejeon, South Korea; 7 Division of Hematology-Oncology, Chonnam National University Hwasun Hospital, Hwasun, South Korea; 8 Department of Internal Medicine, Yeungnam University College of medicine, Daegu, South Korea; 9 Division of Hematology-Oncology, Department of Internal Medicine, Gyeongsang National University College of Medicine, Gyeongsang National University Hospital, Jinju, South Korea; 10 Department of Pathology, Samsung Medical Center, Sungkyunkwan University School of Medicine, Seoul, South Korea

**Keywords:** Ibrutinib, R-CHOP, Epstein-Barr virus-positive, Diffuse large B cell lymphoma

## Abstract

Epstein-Barr virus (EBV) positivity in diffuse large B cell lymphoma (DLBCL) provokes a critical oncogenic mechanism to activate intracellular signaling by LMP1. LMP1 specifically mimics the role of BTK-dependent B cell receptor. Therefore, a trial considering RCHOP therapy along with ibrutinib (I-RCHOP) in combination was conducted among patients with EBV-positive DLBCL. This study was an open-label, single-arm, prospective multicenter phase II clinical trial. Patients received 560 mg of ibrutinib with RCHOP every 3 weeks until 6 cycles were completed or progression or unacceptable toxicity was observed. The primary endpoint was objective response, while secondary endpoints included toxicity, progression-free survival, and overall survival. A matched case-control analysis was completed to compare the efficacy and toxicity of I-RCHOP and RCHOP, respectively, in EBV-positive DLBCL patients. From September 2016 to August 2019, 24 patients proven to have EBV-positive DLBCL in the tissue were enrolled and received I-RCHOP. Their median age was 58 years (range, 28–84 years). The objective overall response was 66.7%, including 16 patients who achieved complete response after 6 cycles. Patients aged younger than 65 years presented a superior OR (87.5%) as compared with those older than 65 years (25.0%; *p* = 0.01). In a matched case-control study, I-RCHOP therapy provoked a more favorable complete response rate (87.3%) than did RCHOP (68.8%) in those younger than 65 years. Treatment-related mortality was linked most frequently with I-RCHOP therapy (four patients presented with unusual infection without Gr3/4 neutropenia) in the older age group (age ≥ 65 years). In conclusion, in this phase II trial for EBV-positive DLBCL, I-RCHOP was effective but did not show a significant improvement in response and survival in comparison with RCHOP. Also, I-RCHOP promoted serious toxicity and treatment-related death in older patients.

**Electronic supplementary material:**

The online version of this article (10.1007/s00277-020-04005-6) contains supplementary material, which is available to authorized users.

## Introduction

Epstein-Barr virus (EBV)-positive, diffuse large B cell lymphoma (DLBCL) is an EBV-positive, monoclonal, large B cell lymphoproliferative disorder mainly seen in individuals older than 50 years of age [1, 2]. Previous studies have shown that EBV-positive DLBCL is mainly correlated with high-intermediate/high International Prognostic Index (IPI) scores, advanced disease stage (III/IV) at diagnosis, and inferior outcomes in comparison with EBV-negative DLBCL [3, 4].

EBV-infected cells encode eight EBV-encoded latent genes consisting of six EBV-encoded nuclear antigens (EBNAs) and two latent membrane proteins (LMP1 and LMP2) [5]. LMP1 and LMP2 in particular behave like B cell receptor (BCR)-mediated Akt, which is dependent upon spleen tyrosine kinase and Bruton’s tyrosine kinase (BTK). These tyrosine kinases contribute to enhanced cellular proliferation and survival signaling such as the nuclear factor-kappa B (NF-κB) and phosphoinositide 3-kinase (PI3K/Akt) pathways [6]. In our experiment, we transfected vector-encoding LMP1 or LMP1_delCTAR1(C-terminal-activating region 1) and LMP1_delCTAR2 into a DLBCL cell line and observed the BCR signaling pathway (Supplementary Materials and methods). As a result, the BTK signaling pathway was activated when LMP1 was expressed. It was also confirmed that the CTAR1 of LMP1 was an important domain for BTK activation. In addition, cell viability was decreased when the BTK inhibitor (ibrutinib) was treated with LMP1-expressing cells (Supplementary Fig. S1). Based on previous studies that proved the efficacy of rituximab-cyclophosphamide-doxorubicin hydrochloride-vincristine sulfate-prednisone (RCHOP) therapy combined with ibrutinib (I-RCHOP), we designed this study to boost the anti-tumor effect of EBV-positive DLBCL [7, 8]. Here, we report the results of an open-label, multicenter phase II study that investigated the combination ibrutinib and RCHOP for EBV-positive DLBCL using a matched case-control study.

## Patients and methods

### Study design and treatment

This study was an open-label, single-arm, prospective multicenter clinical trial for evaluating the efficacy and toxicity of I-RCHOP in subjects with newly diagnosed, chemotherapy-naïve, EBV-positive DLBCL. We defined EBV positivity as a finding of 20% or more of EBV-encoded RNA in situ hybridization–positive tumor cells among total tumor cells during pathology evaluation [2]. Ibrutinib (560 mg/day) was administrated orally and RCHOP (rituximab 375 mg/m^2^, cyclophosphamide 750 mg/m^2^, doxorubicin 50 mg/m^2^, vincristine 1.4 mg/m^2^ (maximum total of 2 mg) on day 1 and oral prednisone 100 mg on days 1–5) therapy was administered intravenously every 21 days, respectively, until the end of 6 cycles or progression or unacceptable toxicity were observed.

### Patient eligibility

Patients had to fulfill the following criteria to be included in this research: (1) histologically confirmed EBV-positive DLBCL without central nervous system involvement; (2) aged 19 years or older; (3) no previous treatment for DLBCL, although we allowed the usage of prednisolone 100 mg or equivalent dosage of any type of steroid (for a maximum of 7 days) and radiation for reducing symptoms related with mass effect; (4) one or more measurable lesions; (5) Eastern Cooperative Oncology Group (ECOG) performance status of 0 to 2; and (6) adequate organ function. The exclusion criteria included any life-threatening illness, medical condition, or organ system dysfunction that could compromise the subject’s safety, according to the investigator’s opinion. Written informed consent was obtained from each patient prior to study enrollment. This study was approved by the institutional review board of each institution and conducted in accordance with the tenets of the Declaration of Helsinki. This trial was registered at www.ClinicalTrials.gov as NCT02670616.

### Matched case-control study design

**Fig. 1:**
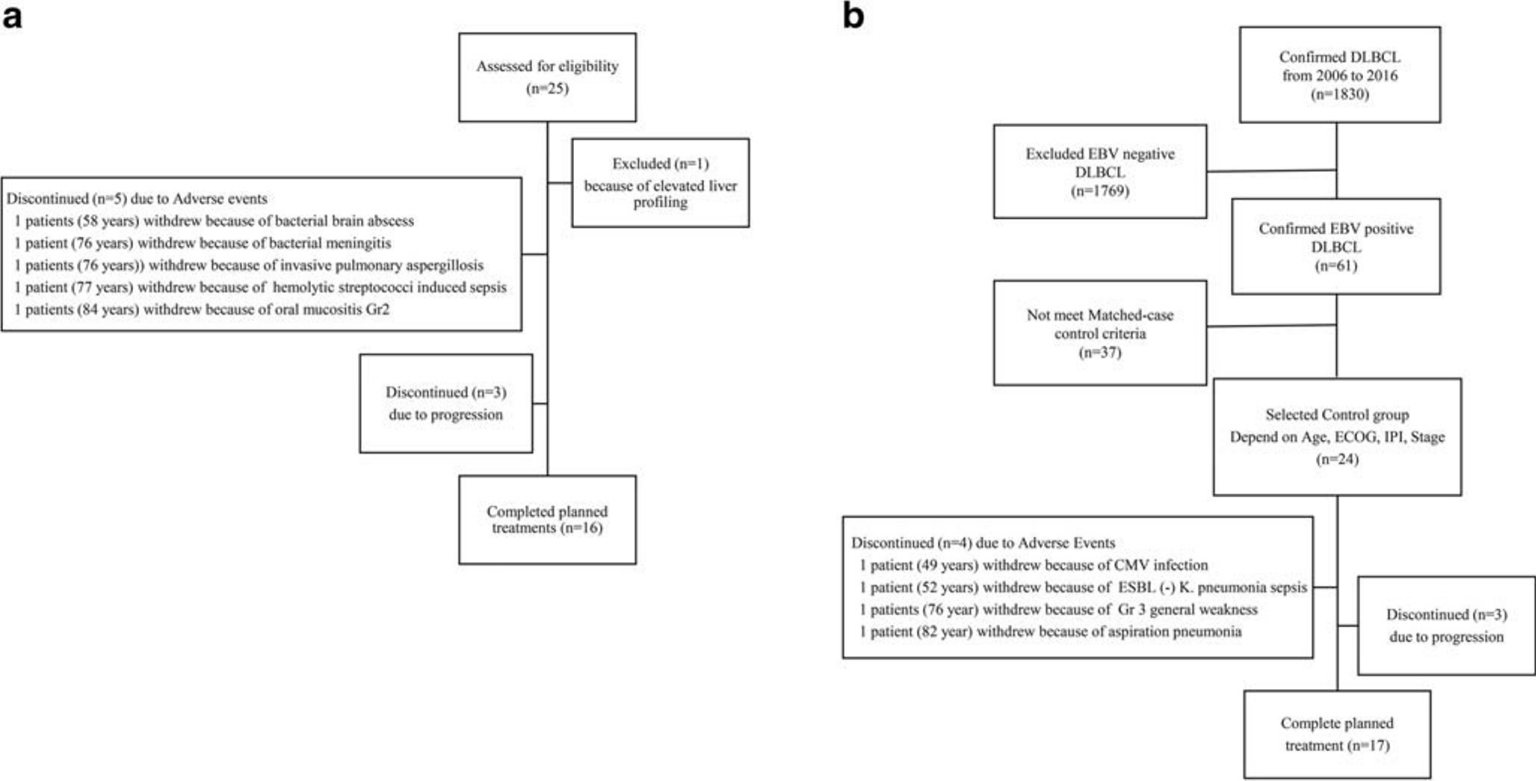
Profiles of patients enrolled in the IVORY study (**a**); Control (RCHOP) group enrolled in a matched case-control study (**b**)

In addition, we organized a case-control study to figure out the superiority (e.g., overall response rate, toxicity) of RCHOP (control) and I-RCHOP (case) in EBV-positive DLBCL. To serve as matched controls, we selected EBV-positive DLBCL patients who received only RCHOP chemotherapy from 2006 to 2018 as part of Samsung Medical Center’s prospective lymphoma registry (Fig. 1b). Especially, we employed strict matching categories, which were age, ECOG performance status, IPI score, and Ann Arbor stage (Supplementary Table S1).

### Study endpoints

The primary endpoint of this research was the objective response rate (ORR) based on the proportion of complete response (CR) and partial response (PR). Secondary endpoints included toxicity, progression-free survival (PFS), and overall survival (OS). Adverse events (AEs) were assessed according to the Common Terminology Criteria for Adverse Events version 4.0.

### Assessment

In study participants, complete blood count, organ function (i.e., creatinine, aspartate transaminase, alanine transaminase), EBV DNA titer, electrocardiogram (ECG), transthoracic echocardiogram (TTE), and measurable lesions were assessed with computed tomography (CT) scans of the neck, chest, and abdominal pelvis or ^18^F-fluorodeoxyglucose positron-emission tomography (PET)/CT scans at before enrollment, during treatment, and at the end of treatment. We assessed response according to the Lugano response criteria for non-Hodgkin’s lymphoma [9].

### Statistical analyses

The aforementioned previous study with EBV-positive DLBCL revealed a 50% complete response rate was achieved by RCHOP without the use of ibrutinib [1]. Separately, the phase I clinical trial with DLBCL patients who received ibrutinib (560 mg/day) in conjunction with RCHOP presented a 95% overall response rate, including both cases of CR and PR [10]. From these results, we hypothesized that the complete response rate of EBV-positive DLBCL might be up to 80% when ibrutinib and RCHOP were combined. Thus, if we could achieve complete response in 14 out of 21 patients, we could rule that our new regimen might be effective for this disorder and likely could start further investigations such as phase I/II trials (P0 = 50%, one-sided alpha = 5%, P1 = 80%, statistical power = 90%) [11]. Considering a 10% rate of interruption, a total of 24 patients were included.

Statistical analyses were performed using the IBM PASW version 24.0 software program (SPSS Inc., Chicago, IL, USA). In the scheme to demonstrate the superiority of I-RCHOP and RCHOP, descriptive statistics was reported as proportions and medians, and intergroup comparisons were achieved using Fisher’s exact test for categorical variables. In addition, survival curves were achieved using the Kaplan-Meier method, and the comparison of survival rates was calculated with the log-rank test. Univariate and multivariate analyses for the assessment of predictive values of I-RCHOP were completed using Cox’s regression model. A two-sided *p* value of less than 0.05 was considered to be statistically significant.

## Results

### Patient characteristics

Between September 2016 and August 2019, 24 patients were enrolled from 10 institutes. The cutoff date for analysis was March 2019, and the median follow-up period was 7.9 months, with six death events (25%). Sixteen patients (67%) finished 6 cycles of chemotherapy. In the 48 patients who were confirmed to have EBV-positive DLBCL at Samsung Medical Center for the purpose of the matched case-control study, baseline clinical characteristics were balanced comparatively among both the I-RCHOP (*n* = 24) and RCHOP (*n* = 24) groups (Table 1). The median ages were 58 years (range, 28–84 years) in I-RCHOP and 57 years (range, 26–82 years) in RCHOP. In both groups, approximately 70% of patients equally presented with high-intermediate/high IPI scores; in addition, more than half patients presented with Ann Arbor stages III or IV.

**Table 1:** Baseline characteristics of EBV-positive DLBCL patients according to I-RCHOP and RCHOP alone

Variable	I-RCHOP (*n* = 24)	RCHOP (*n* = 24)	*p* value
Sex, no. (%)			
Male	17 (70.8)	12 (50.0)	0.24
Female	7 (29.2)	12 (50.0)	
Age			
Median (range)	58 (28**–**84)	57 (26**–**82)	
Older than 60 years	10 (41.7)	10 (41.7)	1.00
Older than 70 years	6 (25.0)	6 (25.0)	1.00
Performance status, no. (%)			
ECOG 0–1	21 (87.5)	21 (87.5)	1.00
ECOG ≥ 2	3 (12.5)	3 (12.5)	
IPI risk group, no. (%)			
Low/low-intermediate	7 (29.2)	6 (25.0)	1.00
High-intermediate/high	17 (70.8)	18 (75.0)	
Histologic subtype, no. (%)			
ABC type	11 (45.8)	9 (37.5)	0.38
GCB type	8 (33.3)	5 (20.8)	
Not evaluated	5 (20.8)	10 (41.7)	
Elevated LDH, no. (%)	19 (79.2)	23 (95.8)	0.19
BM involvement, no. (%)	1 (4.2)	4 (16.7)	0.35
Ann Arbor stage, no. (%)			
I–II	7 (29.2)	6 (25.0)	1.00
III–IV	17 (70.8)	18 (75.0)	
Detection of EBV DNA in serum, no. (%)	8 (33.3)	9 (37.5)	0.18
Patients who finished 6 cycles no. (%)	16 (66.7)	17 (70.8%)	1.00

*ECOG* Eastern Cooperative Oncology Group, *LDH* lactic dehydrogenase, *IPI* International Prognostic Index, *ABC* activated B cell, *GCB* germinal B cell

### Response

The ORR of I-RCHOP was 66.7% (*n* = 16). The most common cause for treatment interruption was AEs (*n* = 5; 21%), followed by disease progression (*n* = 3; 12.5%) (Fig. 1a). Among the I-RCHOP patients, who displayed a 66.7% ORR, those aged younger than 65 years (87.5%) achieved a superior ORR than did those older than 65 years (25.0%; *p* = 0.01; Table 2). When comparing with findings of the matched case-control group, for all patients aged younger than 65 years, though there was a higher CR rate for I-RCHOP (87.3% vs. 68.8%), the ORR (87.5% vs. 75.0%; *p* = 0.69) between I-RCHOP and RCHOP did not show a significant difference. In the patients older than 65 years of age, 50% of patients in the I-RCHOP group dropped out previously due to unacceptable toxicities before the end of the treatment period; thus, the response rate was superior in patients who received RCHOP (50.0% vs. 25.0%; *p* = 0.81; Table 3).

**Table 2:** Response rate according to age group

	Total patients		Age < 65 years		Age ≥ 65 years		*p* value
	*n* = 24		*n* = 16		*n* = 8		
	*n*	%	*n*	%	*n*	%	
I-RCHOP							
Overall	16	66.7	14	87.5	2	25.0	0.01
Complete response	16	66.7	14	87.5	2	25.0	0.01
Progression	3	12.5	1	6.3	2	25.0	
Not evaluable	5	20.8	1	6.3	4	50.0	
RCHOP							
Overall	16	66.7	12	75.0	4	50.0	0.54
Complete response	15	62.5	11	68.8	4	50.0	0.70
Partial response	1	4.2	1	6.3	0	0	
Progression	4	16.7	2	12.5	2	25.0	
Not evaluable	4	16.7	2	12.5	2	25.0

**Table 3:** Response rate according to I-RCHOP and R-CHOP

		I-RCHOP		RCHOP		
		*n* = 24		*n* = 24		
	Response to treatment	*n*	%	*n*	%	*P*-value
Total patients	Overall response	16	66.7	16	66.7	1.00
	Complete response	16	66.7	15	62.5	1.00
	Partial response	0	0	1	4.2	
	Progressive disease	3	12.5	4	16.7	
	Not evaluated	5	20.8	4	16.7	
		*n* = 16		*n* = 16		
		*n*	%	*n*	%	*P*-value
< 65 years	Overall response	14	87.5	12	75.0	0.69
	Complete response	14	87.3	11	68.8	0.53
	Partial response	0	0	1	6.3	
	Progressive disease	1	6.3	2	12.5	
	Not evaluated	1	6.3	2	12.5	
		*n* = 8		*n* = 8		
		*n*	%	*n*	%	*P*-value
≥ 65 years	Overall response	2	25.0	4	50.0	0.81
	Complete response	2	25.0	4	50.0	0.81
	Progressive disease	2	25.0	2	25.0	
	Not evaluated	4	50.0	2	25.0	

### Toxicity

Across I-RCHOP and RCHOP, most patients older than 65 years of age in I-RCHOP group could not complete 6 cycles of chemotherapy (I-RCHOP, 87.5% vs. 25.0%; RCHOP 81.3% vs. 50.0%) due to grades 3/4 AEs (I-RCHOP, 40.0% vs. 82.5%; RCHOP, 75.0% vs. 75.0%). In addition, there were not any EBV reactivation case. In the patients who received I-RCHOP, there were four treatment-related deaths mainly associated with the unusual infection without grades 3/4 neutropenia (Table 4). For example, a patient younger than 65 years died due to a disseminated brain abscess caused by bacteria (confirmed by brain magnetic resonance imaging), while three patients older than 65 years of age died due to bacterial meningitis (diagnosed by cerebrospinal fluid analysis, brain magnetic resonance imaging), sepsis combined with pneumonia caused by streptococci hemolytic species, and invasive pulmonary aspergillosis, respectively.

**Table 4:** Grades 3/4 toxicity profile

	Category	< 65 years		≥ 65 years	
		Case (*n* = 16)	Control (*n* = 16)	Case (*n* = 8)	Control (*n* = 8)
Hematologic	Neutropenia	5 (31.3)	12 (75.0)	4 (50.0)	5 (62.5)
	Neutropenic fever	2 (12.5)	3 (18.8)	1 (12.5)	2 (25.0)
	Anemia	1 (6.25)	1 (6.25)	1 (12.5)	1 (12.5)
	Thrombocytopenia	3 (18.8)	1 (6.25)	4 (50.0)	1 (12.5)
Non-hematologic	Diarrhea	1 (6.25)		1 (12.5)	
	Pneumonia			3 (37.5)^a^	2 (25.0)
	Heart failure		1 (6.25)	1 (12.5)	1 (12.5)
	Sepsis	1 (6.25)	1 (6.25)	1 (12.5)^b^	2 (25.0)
	Brain abscess	1 (6.25)^c^			
	Meningitis			1 (12.5)^d^	

^a^Invasive pulmonary aspergillosis

^b^Hemolytic streptococci-induced sepsis

^c^Bacteria-induced brain abscess

^d^Bacteria-induced meningitis

### Survival

**Fig. 2:**
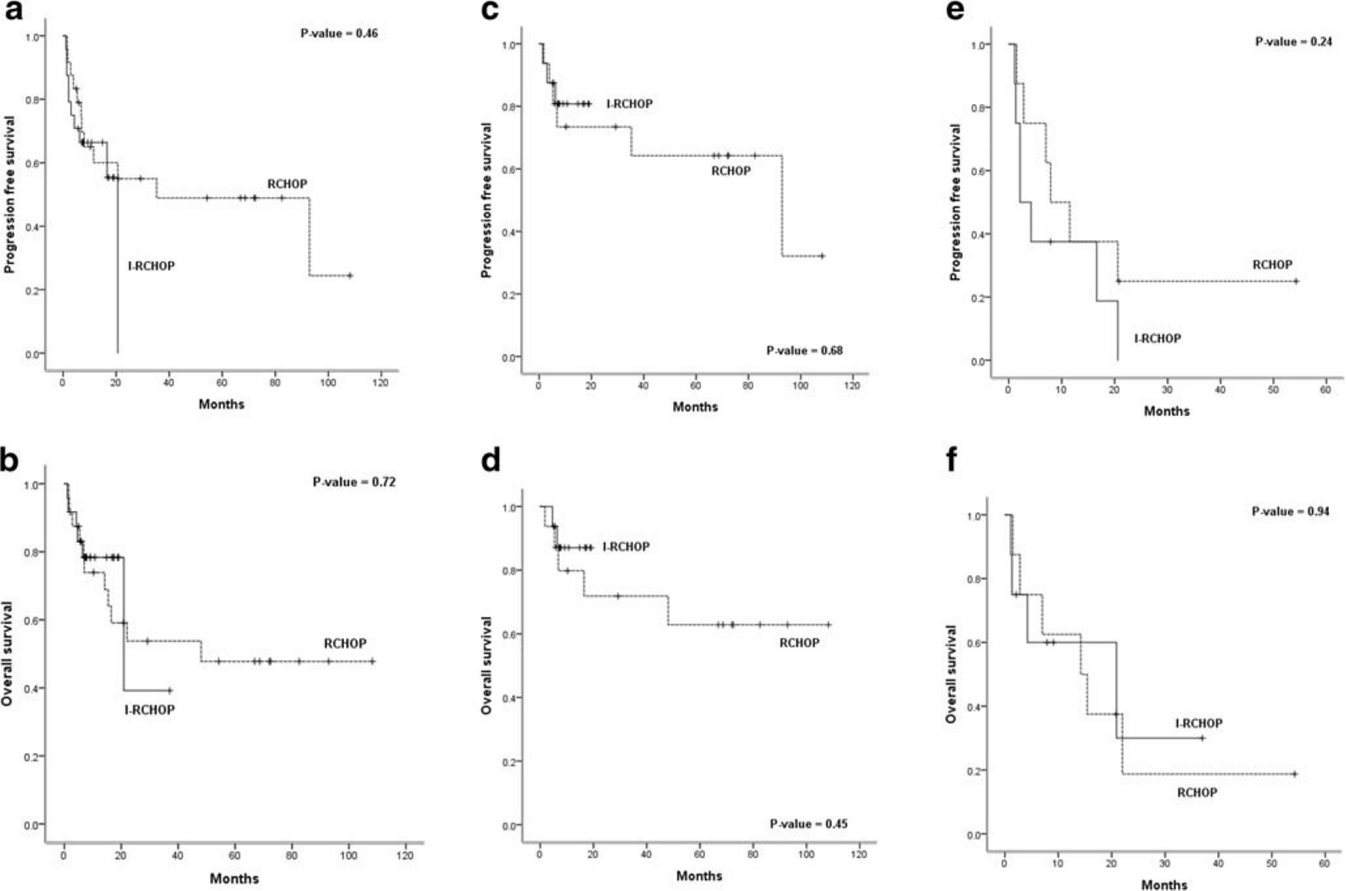
Kaplan-Meier analyses of PFS (**a**) and OS (**b**) according to I-RCHOP and RCHOP in patients with EBV-positive DLBCL; comparison of PFS (**c**) and OS (**d**) of I-RCHOP and RCHOP in patients younger than 65; comparison of PFS (**e**) and OS (**f**) of I-RCHOP and RCHOP in patients older than 65 years

**Fig. 3:**
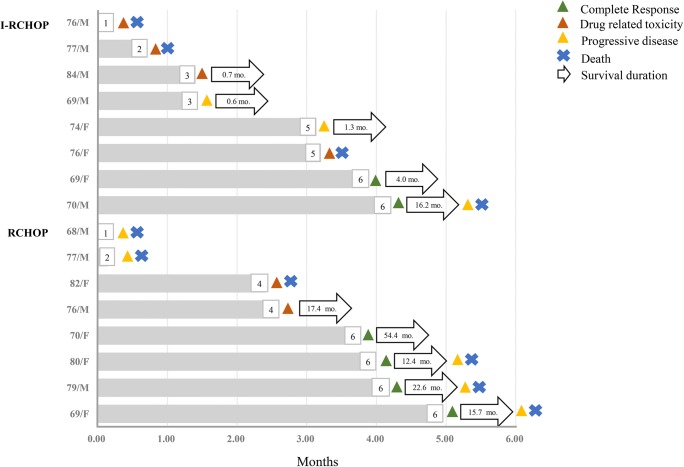
Swimmer plot of patients aged older than 65 years in case and control group

Median PFS (20.6 months, 95% CI non-evaluable vs. 35.3 months, 95% CI 0.0–86.9; *p* = 0.46) and median OS (20.9 months, 95% CI 0.6–41.2 vs. 48.1 months, 95% CI NR; *p* = 0.72) according to I-RCHOP and RCHOP were not significantly different (Fig. 2a and b). Based on the one-year survival analysis, one-year OS (33.3% vs. 58.3%; *p* = 0.15) and PFS (29.2% vs. 36.8%, *p* = 0.24) were not correlated with more favorable outcomes in I-RCHOP than in RCHOP among all patients. In line with age-based case-control comparisons, in patients aged younger than 65 years, the median PFS (NR vs. 92.2 months, 95% CI 9.8–176.0; *p* = 0.68) and OS (NR vs. NR; *p* = 0.45) showed a superior tendency in the I-RCHOP group (Fig. 2c and d). In patients aged older than 65 years, the survival benefit was lower in the I-RCHOP group because of the large number of patients terminated during the initial treatment (Figs. 2e, f, and 3).

### Univariate and multivariate analysis of I-RCHOP

Univariate and multivariate analyses were performed to determine the predictive factors of I-RCHOP from the Cox proportional hazards model analysis of PFS according to baseline characteristics. The results of univariate analysis revealed worse outcomes in those aged older than 65 years (HR 5.54, 95% CI 1.37–22.38; *p* = 0.02) and favorable outcomes in those who finished 6 cycles (HR, 0.03; 95% CI, 0.03–0.21; *p* = 0.00). In multivariate analysis, having completed 6 cycles of chemotherapy was only demonstrated to be an independent prognostic factor for I-RCHOP (HR, 0.02. 95% CI, 0.00–0.21; *p* = 0.00; Table 5).

**Table 5:** Univariate and multivariate analysis of I-RCHOP for estimating prognostic factors

Variable	Univariate			Multivariate		
	HR	95% CI	*p* value	HR	95% CI	*p* value
Male sex	1.03	0.26–4.12	0.97			
Age ≥ 65 years	5.54	1.37–22.38	0.02	0.61	0.12–3.19	0.55
ECOG ≥ 2	1.46	0.66–3.26	0.35			
Stage ≥ 3	1.62	0.34–7.79	0.55			
B-symptom	1.35	0.30–6.07	0.70			
Extranodal involvement	0.45	0.05–3.80	0.46			
IPI ≥ 3	4.24	0.88–20.56	0.07			
Bulky mass ≥ 10 cm	4.34	0.87–21.74	0.07			
Finished 6 cycles	0.03	0.03–0.21	0.00	0.02	0.00–0.21	0.00

*ECOG* Eastern Cooperative Oncology Group, *IPI* International Prognostic Index

## Discussion

The previous reported studies about EB-positive DLBCL have suggested that EBV could encourage an inferior outcome in DLBCL [1–4]. EBV using the host’s BCR-mediated protein tyrosine kinase can prevent apoptosis and stimulate the proliferation of infected cells, leading to lymphomagenesis [5, 12, 13]. Based on previous findings, we attempted to combine ibrutinib and RCHOP to improve the outcome of EBV-positive DLBCL. The current study (IVORY) revealed a limited improvement in response and survival in those aged younger than 65 years, while the treatment was associated with severe toxicity in those aged older than 65 years. Although EBV positive was expected to be a potent marker to predict reponse of BTK inhibitor, the therapeutic efficacy of I-RCHOP in the real world showed less than expected. Thus, it was figured out that being EBV-positive was not enough to be a prognostic marker of the BTK inhibitor in the real world.

In our study, including a comparison of I-RCHOP versus RCHOP for EBV-positive DLBCL, the response and survival outcomes cannot support the superiority of I-RCHOP. In patients over the age of 65 years, I-RCHOP showed a lower response rate due to the high rate of early termination (50.0%) caused by severe drug-related toxicity such as sepsis, brain abscess, and meningitis. However, in the age group of those younger than 65 years, I-RCHOP achieved a higher CR as a consequence survival curve of I-RCHOP was presented above that of RCHOP. Although caution should be taken when interpreting our results due to the small number of patients in our findings, as reported by Cox proportional hazards model analysis of PFS, I-RCHOP led to a better prognosis in patients younger than 65 years and with the completion of 6 cycles of chemotherapy (Table 4). Elsewhere, a randomized phase III study (PHOENIX) evaluated I-RCHOP in non-GCB DLBCL. The younger patients (aged < 60 years) with I-RCHOP obtained superior EFS, PFS, and OS as compared with RCHOP plus placebo, and older patients (aged > 60 years) experienced early discontinuation of I-RCHOP chemotherapy due to rates of severe AEs and worse outcomes [14]. These results imply that the patient’s age should be taken into consideration before applying I-RCHOP. For younger patients with non-GCB or EBV-positive DLBCL, ibrutinib could be considered as one of the methods to improve survival.

Park et al. demonstrated that non-neutropenic opportunistic infection after the administration of RCHOP might be associated with the depletion of B cells and impaired humoral immune due to repeated doses of rituximab [15]. The most frequent causes of fever without neutropenia were interstitial pneumonia without bacteremia followed by catheter-related infection mainly occurring at 4 cycles in. In addition, causes of opportunistic infection included *Candida*, *Pneumocystis jirovecii*, *Cytomegalovirus*, and *Mycobacterium avium*.

Among 24 patients who received I-RCHOP in this study, four experienced severe infection in absence of fever without neutropenia before 4 cycles of chemotherapy (Supplementary Table S2). There was one case of invasive pulmonary aspergillosis, which was not observed in the control group (Fig. 1). Aspergillosis that was only seen in patients older than 60 years of age in PHOENIX trial was reported in six cases in I-RCHOP group, but it also occurred in 2 patients received RCHOP [16, 17]. The previous studies showed that the elimination of aspergillosis occurs by phagocytosis involving the Toll-like receptor 9-BTK-calcineurin-nuclear factor of T cell pathway [18]. Inhibiting the BTK pathway suggests that it could lead to impairment in the adaptive and innate immune systems with fungal infection. However, the recent study reported the most infections during ibrutinib treatment are bacterial and viral infection [19].

In a pooled analysis of 4 randomized trials in CLL and MCL, the reported incidence of fungal infection in ibrutinib was 7%, which was similar to it in comparator arm (6%) [20]. Therefore, we should consider prophylaxis according to standard of care in subjects who are at increased risk for opportunistic infections.

In conclusion, I-RCHOP, which was proposed as a method to avoiding a worse outcome of EBV-positive DLBCL, did not yield a better outcome than RCHOP.

## Electronic supplementary material

Supplementary Fig. S1. Western blot analysis of BTK, PLCγ2, and NF-κB phosphorylation, according to LMP1. Riva cells were transient with vector encoding LMP1 or LMP1_delCTAR1(C-terminal-activating region 1) and LMP1_delCTAR2. Actin was included as a loading control (**a)**; Riva cells were transient with indicated vectors and treated with the indicated doses of ibrutinib for 72 hr, followed by the CCK-8 assay. Each experiment was performed with triplicate samples. *P*-values were determined by one-way repeated-measures ANOVA. The double asterisk indicates a statistically significant difference at *P* ≤ 0.01, one asterisk significant at *P* ≤ 0.05 (**b)**. (PNG 199 kb).

High resolution image (TIF 96 kb).

ESM 1 (DOCX 20 kb).

ESM2 (DOCX 13 kb).
