# Supplementary figures and images for: A phase II study of ibrutinib in combination with rituximab-cyclophosphamide-doxorubicin hydrochloride-vincristine sulfate-prednisone therapy in Epstein-Barr virus-positive, diffuse large B cell lymphoma (54179060LYM2003: IVORY study): results of the final analysis
Source: Ann Hematol. 2020 Apr 24;99(6):1283–91. doi: 10.1007/s00277-020-04005-6 (PMC7237534; doi:10.1007/s00277-020-04005-6)

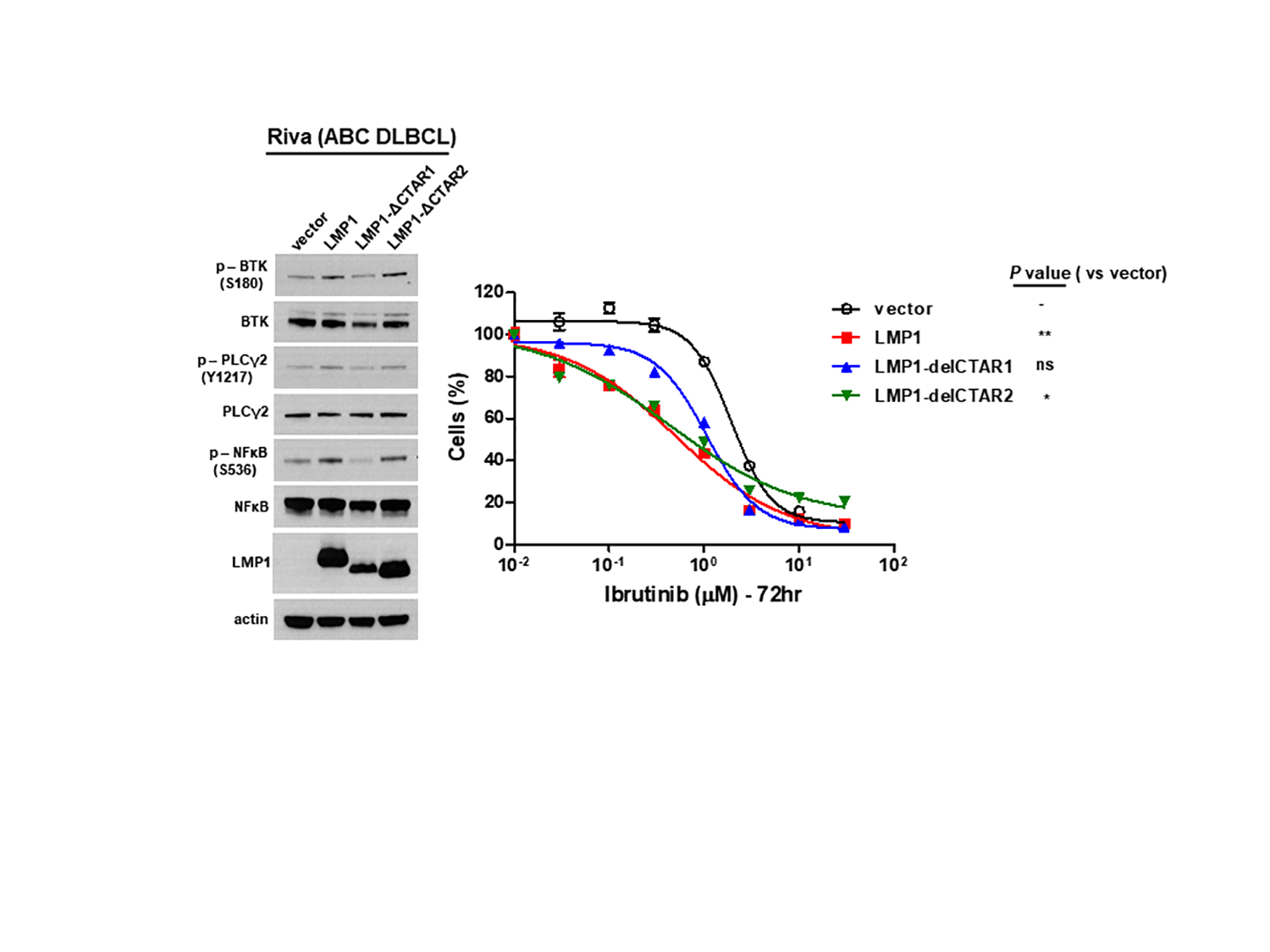

Supplement: Supplementary file 1 — Western blot analysis of BTK, PLCγ2, and NF-κB phosphorylation, according to LMP1. Riva cells were transient with vector encoding LMP1 or LMP1_delCTAR1(C-terminal-activating region 1) and LMP1_delCTAR2. Actin was included as a loading control (a); Riva cells were transient with indicated vectors and treated with the indicated doses of ibrutinib for 72 hr, followed by the CCK-8 assay. Each experiment was performed with triplicate samples. P-values were determined by one-way repeated-measures ANOVA. The double asterisk indicates a statistically significant difference at P ≤ 0.01, one asterisk significant at P ≤ 0.05 (b). (PNG 199 kb). [file 277_2020_4005_Fig1_ESM.png]

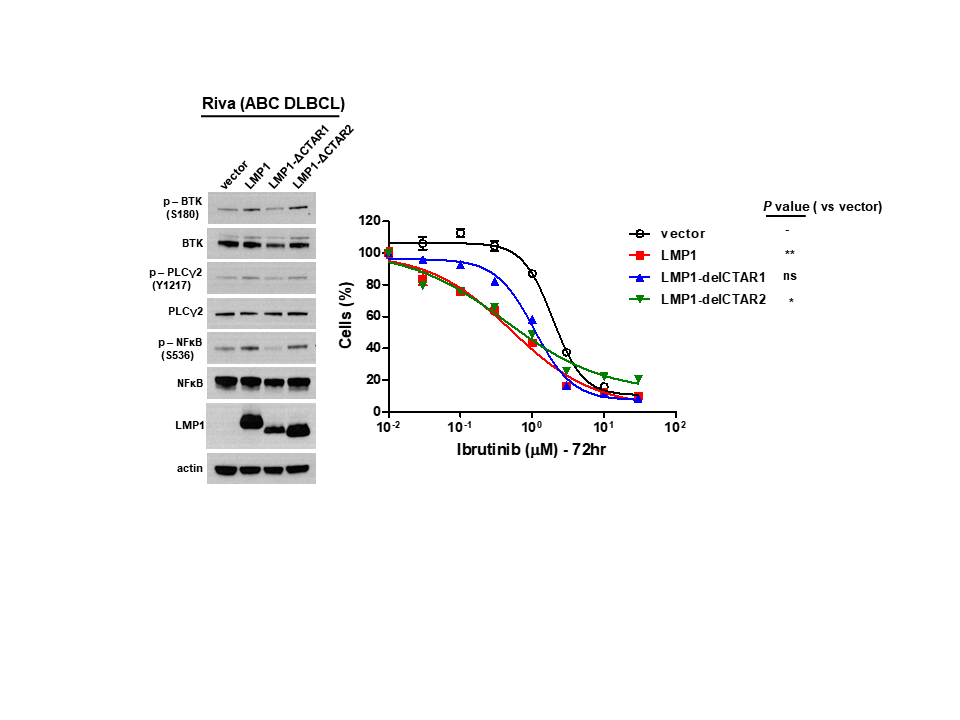

Supplement: Supplementary file 2 — High resolution image (TIF 96 kb). [file 277_2020_4005_MOESM1_ESM.tif]
