# Supplementary material for: A phase II study of ibrutinib in combination with rituximab-cyclophosphamide-doxorubicin hydrochloride-vincristine sulfate-prednisone therapy in Epstein-Barr virus-positive, diffuse large B cell lymphoma (54179060LYM2003: IVORY study): results of the final analysis
Source: Ann Hematol. 2020 Apr 24;99(6):1283–91. doi: 10.1007/s00277-020-04005-6 (PMC7237534; doi:10.1007/s00277-020-04005-6)
Supplement: Supplementary file 3 — (DOCX 20 kb). [file 277_2020_4005_MOESM2_ESM.docx]

**Supplementary Table S1** Matched case–control criteria according to age stratification

| Median age (years) | I-RCHOP | RCHOP | |
| --- | --- | --- | --- |
|  | Case group | Total | Selected control group |
|  | *n* = 24 | *n* = 61 | *n* = 24 |
| < 50 | 6 | 15 | 6 |
| 51–55 | 4 | 5 | 4 |
| 56–60 | 4 | 5 | 4 |
| 61–65 | 2 | 8 | 2 |
| 66–70 | 3 | 9 | 3 |
| 71–75 | 1 | 12 | 1 |
| ≥ 76 | 4 | 7 | 4 |

**Supplementary Table S2**

Absolute neutrophil count of patients who did not complete chemotherapy.

| Age/sex | No. of cycles | Absolute neutrophil count (/µL) at last cycle | | | | | |
| --- | --- | --- | --- | --- | --- | --- | --- |
|  |  | Baseline | 1^st^ | 2^nd^ | 3^rd^ | 4^th^ | 5^th^ |
| 76/M | 1 | 8,510 | 8,510 |  |  |  |  |
| 77/M | 2 | 4,200 | 4,745 | 1,576 |  |  |  |
| 58/F | 4 | 3,620 | 3,690 | 25,370 | 22,160 | 8,460 |  |
| 76/F | 5 | 2,350 | 2,940 | 11,230 | 8,460 | 12,170 | 5,180 |
