# Supplementary material for: A phase II study of ibrutinib in combination with rituximab-cyclophosphamide-doxorubicin hydrochloride-vincristine sulfate-prednisone therapy in Epstein-Barr virus-positive, diffuse large B cell lymphoma (54179060LYM2003: IVORY study): results of the final analysis
Source: Ann Hematol. 2020 Apr 24;99(6):1283–91. doi: 10.1007/s00277-020-04005-6 (PMC7237534; doi:10.1007/s00277-020-04005-6)
Supplement: Supplementary file 4 — (DOCX 13 kb). [file 277_2020_4005_MOESM3_ESM.docx]

**Supplementary Materials and methods**

**Cell lines, culture conditions, transfection, and inhibitors**

Riva cells were purchased from Leibniz-Institut DSMZ-Deutsche Sammlung von Mikroorganismen und Zellkulturen GmbH (Braunschweig, Germany). Cell lines were cultured in RPMI-1640 medium supplemented with 10% heat-inactivated fetal bovine serum (FBS), penicillin, and streptomycin (Gibco-BRL, Grand Island, NY, USA) in a humidified 5% CO_2_ atmosphere. Expression plasmids encoding wild-type (pTag4C-LMP1) and mutant LMP1 (LMP1_delCTAR1 and LMP1_delCTAR2) were obtained from Dr. Y. Cao (Central South University, Changsha, China). Cells were transiently transfected using the Amaxa electroporation system (Amaxa, Gaithersburg, MD). Ibrutinib was purchased from Selleck Chemicals (Houston, TX77054).

**Assessment of cell viability**

Drug effects on cell viability were monitored using the Cell Counting Kit-8 reagent (CCK-8) viability assay. For CCK-8 assays, cells were incubated in triplicate in a 96-well plate (final volume, 0.1 mL) in the presence or absence of the indicated test samples, followed by addition of 20 μL of CCK-8 reagent (Dojindo Laboratories, Kumamoto, Japan) to each well. After a 2-hour incubation at 37°C, optical density (OD) at 450 nm was measured using a 96-well multiscanner autoreader. Cell viability was expressed as a percentage (OD of the experimental sample/OD of control).

**Antibodies for Western blotting**

The antibodies employed included those specific for p-BTK(S180), BTK, p-PLCγ2, PLCγ2, p-NF-κB (Cell Signaling, Beverly, MA), NF-κB (Santa Cruz, CA), LMP1 (Dako, Denmark), β-actin (Sigma St. Louis, MO, USA) was used as a loading control. All primary antibodies were diluted to 1:1000, and secondary antibodies were diluted to 1:3000.”
